# Wasting and short-term outcomes among children with cancer in resource-limited settings: A prospective study in Uganda

**DOI:** 10.1371/journal.pone.0330107

**Published:** 2025-08-07

**Authors:** Richard Nyeko, Jaques van Heerden, Joyce Balagadde Kambugu, Fadhil Geriga, Racheal Angom, Teresa de Rojas, Anouk Neven

**Affiliations:** 1 Department of Pediatric and Child Health, Faculty of Medicine, Lira University, Lira, Uganda; 2 Pediatric Oncology Service, Uganda Cancer Institute, Kampala, Uganda; 3 Department of Pediatric Hemato-Oncology, Antwerp University Hospital, University of Antwerp, Antwerp, Belgium; 4 Arztpraxis Mylife, Fulenbach, Solothurn, Switzerland; 5 Luxembourg Institute of Health, Competence Center for Methodology and Statistics, Strassen, Luxembourg; Federal University of Agriculture Abeokuta, NIGERIA

## Abstract

**Background:**

Wasting contributes to poor treatment outcomes in children with cancer, especially in low-resource settings. In these settings, there is inadequate routine, systematic assessment of the wasting status of children with cancer. Wasting is diagnosed based on visual evidence, with a subjective bias for recognition. This study determined the prevalence of wasting at diagnosis among children with cancer at the Uganda Cancer Institute (UCI) and the diagnostic accuracy of “visible wasting” in identifying children with wasting as measured by anthropometric indices, and identified predictors of 6-months negative outcomes.

**Methods:**

We assessed the wasting status at diagnosis, diagnostic accuracy of visible wasting, and 6-month outcomes of children newly diagnosed with cancer at the UCI (both ambulatory and hospitalized) between April 2022 and March 2023. Data were analyzed using SPSS version 26. Descriptive, bivariate, multivariate, and survival analyses were performed as appropriate. Statistical significance was determined at P-value<0.05.

**Results:**

One hundred forty-four children with cancer, with a median age of 10.0 years (interquartile range [IQR] 4.0–14.0 years), were included. The majority, 89 (61.8%), had solid tumor, whereas 55 (38.2%) had hemato-lymphoid malignancies. Thirty-two (22.2%) of the participants had visible wasting, and 57 (39.6%) were wasted based on anthropometric measurements, 32 (56.1%) of whom showed no visible wasting. Visible wasting had a low sensitivity of 43.9% (95% CI 30.7–57.6) – ROC 0.32 (95% CI 0.23–0.42), with a false negative rate of 56.1%. Overall, visible wasting missed up to 80.6% (25/31) of children with moderate wasting and 26.9% (7/26) with severe wasting. Twenty-one (14.6%) of the patients died, 8 (38.1%) of whom were deemed to be wasted, and 15 (71.4%) had anthropometrically-defined wasting. Neutropenia occurred in 20.8% (n = 30) of the participants and sepsis in 13.9% (n = 20). In univariate analyses, wasted patients were more likely to develop neutropenia (OR 3.63; 95% CI 1.56–8.42; p = 0.003), sepsis (OR 4.50; 95% CI 1.65–12.29; p = 0.003), and die (OR 3.08; 95% CI 1.15–8.28; p = 0.026).

**Conclusion:**

Wasting at diagnosis is a common problem among children with cancer in this resource-limited setting and is associated with increased risks of neutropenia, sepsis, and mortality. Reliance on visible wasting as a marker for wasting misses other wasted children, some of who may be malnourished and at risk of poor outcome. For accurate categorization of wasting, all patients should undergo a standard anthropometric evaluation.

## Introduction

Wasting, which encompasses nutritional deficiency, is a prevalent problem in low- and middle-income countries (LMICs) [1], responsible for one million deaths per year in children younger than five years [2]. Children with cancer in LMICs are thus more likely to have underlying baseline wasting even before cancer develops, further exacerbating the problems of wasting during cancer management. It is estimated that 46–70% of children with cancer in LMICs have wasting at diagnosis [3–5] – which can be as a result of lack of food (nutritional wasting) [6] or the cancer cachexia process or a combination of both. Wasting has been linked to adverse outcomes during the cancer treatment and survivorship, including increased morbidity, inferior treatment tolerance and response [7,8], and poor clinical outcomes [9–12].

The importance of wasting in childhood cancer therapy, however, remains an underrated issue, especially in low-resource settings, and the prevalence of wasting due to nutritional deficiency among childhood cancer patients is believed to be underestimated [13,14]. Moreover, child wasting in these settings remains largely a ‘hidden problem.’ In Uganda, 46% of the households are moderately to severely food insecure – 30.7% of which are severely food insecure [15]. Relatedly, 29% of children under five are stunted or are moderately wasted [16] – statuses which are difficult to identify without regular assessment. Routine and regular assessment, early identification, and monitoring for wasting and the potential nutritional deficits across the cancer care spectrum are critical to ensuring that appropriate interventions are provided to those that may have nutritional deficits [17].

Anthropometric assessment has been the most widely used method for assessing wasting in children, based on weight, height, mid-upper-arm circumference (MUAC), triceps skinfold thickness (TSFT), and many other measurements. While there is no universally accepted algorithm for assessing wasting in children with cancers [17,18], the International Society of Pediatric Oncology Committee on Pediatric Oncology in Developing Countries (SIOP-PODC) Nutrition Working Group recommends a standardized method of assessment in children with cancer in LMICs based on weight, height, mid-upper-arm circumference (MUAC), and body mass index (BMI), along with a directed clinical examination for signs of inadequate intake and micronutrient deficiencies [19] that may be responsible for such wasting.

In many situations in resource-limited settings, Uganda included, weight is the only measure consistently taken [20,21], mainly for the purpose of medication dosing and not assessing whether the child has wasting or not. Consequently, the diagnosis of wasting is frequently evaluated based only on clinical recognition by observation of visible signs of wasting [22,23]. This means that attention is frequently focused on children who are deemed severely wasted based on the presence of visible severe wasting, defined as the presence of muscle wasting in the gluteal region, loss of subcutaneous fat, or prominence of bony structures, particularly over the thorax [24]. While this may be indicative of obvious wasting, therein lies the subjective risk of missing other wasted children, especially those with moderate degrees of wasting.

Our extensive bibliographic search suggests a paucity of data on wasting and how it impacts treatment outcomes of children and adolescents with cancer in Uganda. Likewise, there is a dearth of literature on how visible wasting, which has traditionally been relied on, relates to anthropometric-defined wasting of children and adolescents with cancer. This study determined the prevalence of wasting at diagnosis among children with cancer at the Uganda Cancer Institute (UCI) and the diagnostic accuracy of “visible wasting” in identifying children with wasting as measured by anthropometric indices, and identified predictors of 6-months negative outcomes.

## Methods

### Study design and setting

This was a prospective cohort study involving children and adolescents diagnosed with cancer aged six months to 17 years treated at the Uganda Cancer Institute (UCI), Uganda, between December 2021 and October 2022.

The study was conducted in the pediatric oncology unit at the UCI in Uganda. UCI is a 200-bed national reference cancer treatment facility, 43 of which are dedicated to children and adolescent inpatients. Approximately 80% of children with cancer in Uganda are treated at the UCI, where about 400–500 new childhood cancer cases are seen annually, making this a representative site in the country for conducting the study. All cancer types are treated at the study setting based on different disease-specific protocols that encompass multimodality treatments that include chemotherapy, surgery, radiation therapy, and supportive therapies—depending on the cancer type. The center is able to provide most of the chemotherapeutic agents with the exception of immunotherapy and some targeted therapies. At the time of this study, children with wasting attributed to nutritional deficiency accessed nutritional interventions from a nearby nutritional unit (Mwanamugimu Nutrition Unit)—located less than 300 meters from the cancer treatment center.

### Study population and sample size estimation

Children and adolescents newly diagnosed with cancer of any type and aged six months to 17 years receiving cancer care at the facility during the study period – both ambulatory and hospitalized, were included in the study. All the children were enrolled at the time of cancer diagnosis. Children who had received prior chemotherapy before referral to the UCI, and those whose caregivers declined to give informed consent for participation in the study were excluded.

#### Sample size estimation for the primary outcome.

The sample size was estimated using the formula for cross-sectional studies by Leslie Kish (1965) [25] (N = Z^2^ P (1-P)/D2) with a standard normal value corresponding to a 95% confidence interval (1.96), absolute errors between the estimated and true value of 8%, and an estimated prevalence of wasting among children with cancer of 34.6%—the rate among childhood cancer patients reported by Ndayisenga et al. (2021) in South West Uganda [26]. The estimated sample size of 136 was adjusted for an approximate dropout rate of 5%, giving a total of 144.

#### Sample size estimation based on the secondary outcomes.

The sample size was calculated based on the formula for diagnostic accuracy studies using an online calculator at https://wnarifin.github.io/ssc/sssnsp.html, based on an assumed sensitivity and specificity of visible wasting of 54% and 96%, respectively, among non-cancer patients in a Kenyan study [27], and an estimated prevalence of wasting among children with cancer of 30%, a precision of 15%, and a standard normal value corresponding to a 95% confidence interval (1.96), giving a sample size of 143 (S1 File).

Therefore, a sample size for the primary objective of 144 was used.

### Study instrument

We used a structured questionnaire which was developed by the investigators in line with the study objectives to collect quantitative data—sectioned into a) baseline information at diagnosis (socio-demographic, clinical, and anthropometric information) and b) short-term (six months) clinical outcome evaluation during cancer treatment. The questionnaire was pre-tested and the necessary improvements were made before being used.

### Sampling and study procedures

The study participants were selected by consecutive enrolment until the required sample size was reached. The caregiver of each identified child was approached and interviewed by the study clinician using an interviewer-administered questionnaire after obtaining consent. The caregivers provided information on the child’s socio-demographic characteristics, including age, sex, number of siblings, and duration of illness, as well as their own (i.e., caregiver’s) socio-demographic information (age, sex, education level, and occupation). Further information was obtained from the child’s medical records regarding the disease characteristics, including the type of cancer, laboratory parameters, and outcome variables. The study participants were then followed up for six months as per standard clinical practice, and we collected vital status and laboratory variables (complete blood count), except if they died or got lost to follow-up and could thus not be traced before the six months elapsed.

### Nutritional status assessment and categorization

#### Visual-based clinical nutritional assessment.

For the traditional visual-based assessment of wasting based on the presence of visible wasting, we relied on the independent clinical records of the primary attending clinicians to decide whether they had diagnosed wasting and hence deemed that the child was wasted or not. This was a routine clinical assessment practice at the time at UCI and was thus done without actively asking the clinicians or influencing their decisions to avoid bias. These clinicians, who were the first to attend to the patients before enrolment into the study, were not part of the study team and were not aware of the study’s objectives.

#### Anthropometric assessment.

For comparison, the wasting status of each participant was determined at baseline based on anthropometric assessments, including measurements of weight in kilograms (kg), height in centimeters (cm), and mid-upper-arm circumference (MUAC) in cm. Weight was measured using the Seca Weighing Scale, weighing up to 20 kg for children under two years of age and up to 160 kg for older children, and recorded to the nearest 0.1 kg. Height or length (for children less than 24 months of age) was measured using a height board (positioned upright for height and horizontally for length) to the nearest 0.1 cm. MUAC was measured on the less active arm (usually the left arm) using a color-coded MUAC tape or non-stretchable measuring tape and recorded to the nearest 0.1 cm. From the above measurements, anthropometric indices were calculated, and the World Health Organization (WHO) standard z-scores were used to determine the wasting status and category of the participant. These included the weight-for-height/length (WFH/L) z-score, MUAC z-score, and BMI-for-age for children aged 5–17 years.

Wasting was defined and classified according to WHO standards [28]. Using this standard, a child was considered to have wasting if the WFH/L z-score (for children <5 years), BMI-for-age z-score (for children 5–17 years), or MUAC z-score were < −2 SD below the median for age and gender. A child was classified as having severe wasting if the respective z-score was < −3SD, moderate wasting if the z-score was between −3SD and −2SD, and normal status if the z-score was > -2SD.

### Laboratory assessments

A basic laboratory test, which included a complete blood count (CBC), was performed at diagnosis (the baseline) and during treatment to assess for chemotherapy-associated toxicities. We also determined the baseline serum albumin level at diagnosis.

#### Definition of short-term outcomes.

Short-term outcomes within six months of diagnosis of childhood cancer were handled as binary endpoints and entailed the following:

Febrile neutropenia was defined according to the Common Terminology Criteria for Adverse Events (CTCAE) as: “A disorder characterized by an ANC <1000/mm^3^ and a single temperature of >38.3 degrees C (101 degrees F) or a sustained temperature of ≥38 degrees C (100.4 degrees F) for more than one hour” [29].

Sepsis was defined clinically as a life-threatening condition caused by a dysregulated host response to infection, characterized by temperature dysregulation and symptoms of other organ dysfunctions.

Mortality in the context of this study was taken as death from any cause during the course of the children’s cancer treatment.

To estimate survival rates, overall survival (OS) was defined as the time duration from the date of cancer diagnosis to death from any cause or to the date the patient was last known to be alive.

### Statistical analysis

Data were entered, cleaned, and analyzed using the Statistical Package for Social Scientists (SPSS) version 26 software package. In univariate analysis, categorical variables were summarized as absolute frequencies and proportions, continuous variables as means and standard deviation (SD) if normally distributed, and median (interquartile range) if the variable was not normally distributed. The prevalence of wasting at diagnosis was calculated as the number of children with wasting as assessed by anthropometric measurements at diagnosis divided by the number of children enrolled in the study. The sensitivity (Se) and specificity (Sp) of the clinical visual assessment of wasting were determined with their 95% confidence intervals (CI) using the anthropometrically derived wasting status as the reference measure. Agreement between the two assessments of wasting was also evaluated by Cohen’s kappa with a 95% CI. Survival rates were stratified by wasting status (wasted or not wasted) based on the two methods of assessment and estimated using the Kaplan-Meier method. Comparisons were based on the log-rank test [30]. Patients alive at the end of the period under consideration for the survival analysis or at the last follow-up date were censored.

In the bivariate analysis, binary logistic regression was used to test for the association between the wasting status at diagnosis as an outcome and the explanatory variables. Multivariate logistic regression analysis was used to determine the factors that were independently associated with the presence of anthropometrically defined wasting and visible wasting at diagnosis. Tests of significance were two-sided. All variables with a p-value <0.2 at bivariate analysis were entered into the multivariate model. The predictor variables included socio-demographic characteristics, the type of cancer diagnosis, and the duration of symptoms. Odds ratios with a 95% CI were used to measure the strength of the association between the outcome and predictor variables.

Likewise, logistic regressions were performed to test the association between the short-term outcomes as the dependent variables and wasting status as explanatory variables. A p-value <0.05 was considered for statistical significance. There was no adjustment for multiplicity, and regression analyses were restricted to individuals with complete data for the variable of interest.

### Ethics considerations

All methods were carried out in accordance with relevant guidelines and regulations, and the study was conducted in accordance with the Declaration of Helsinki. The study was approved by the Uganda Cancer Institute Research and Ethics Committee (UCIREC02/11/2021). Voluntary written informed consent was obtained from the parents or guardians of the children before participating in the study after an explanation of the nature and purpose of the study and the potential benefits and risks, if any. Written informed assent was obtained from children aged 8–17 years old with parental or guardian consent. Participants’ privacy and confidentiality were observed throughout the study. Participants found to be severely wasted and believed to have nutritional deficiency were linked to the nutrition unit in Mulago National Referral Hospital within the same vicinity for nutritional rehabilitation.

## Results

### Description of study participants

**Fig 1:**
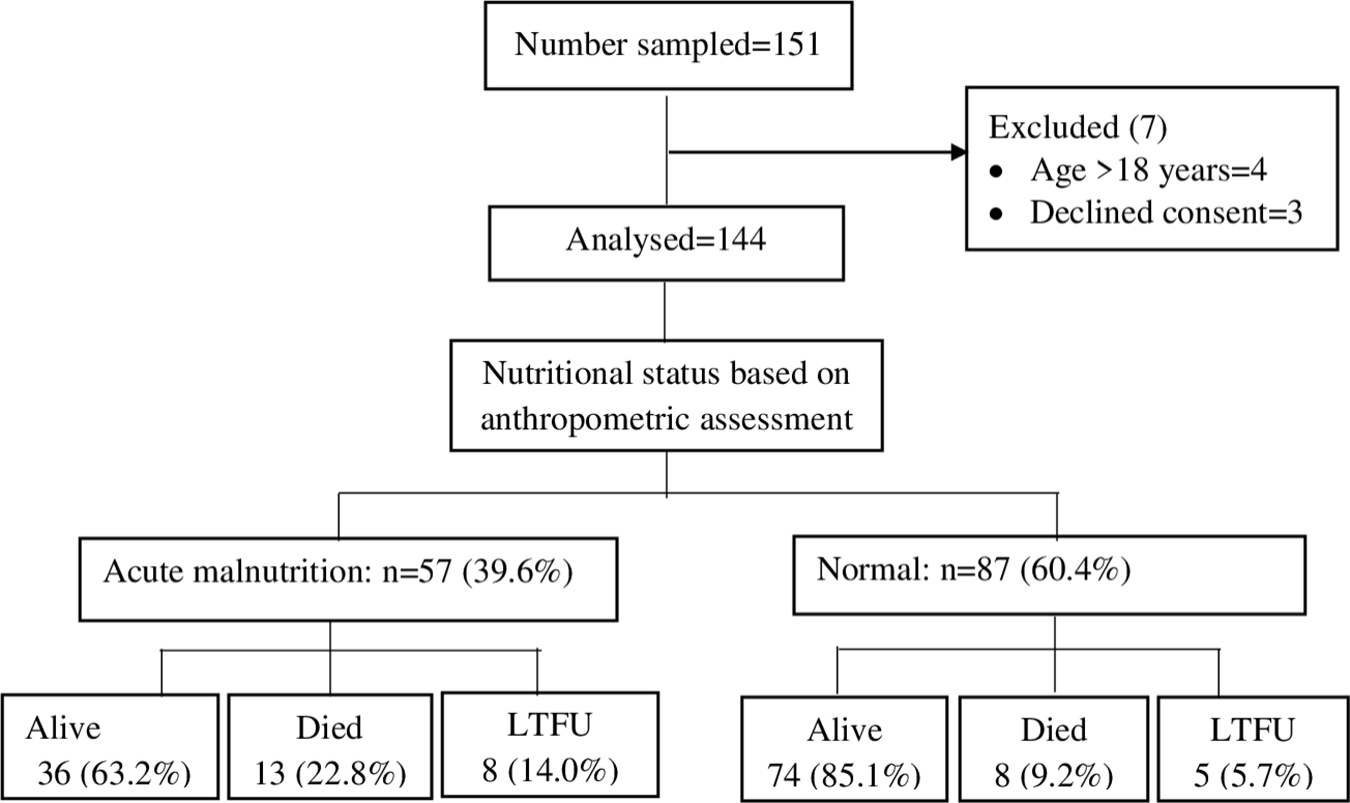
Study flow diagram.

One hundred forty-four children and adolescents newly diagnosed with cancer were enrolled in the study (Fig 1).

The majority were males (56.3%), aged five years and older (72.9%), with a median age of 10.0 years (IQR 4.0–14.0 years). The majority of the participants waited 3 months or more before presenting to the health facility, with a median time to presentation of 4.0 months (IQR 3.0–9.0). Over one-third had hematological malignancies, while slightly more than half had other solid tumors, and only a small proportion had brain tumors (Table 1). The majority of the children were being cared for by their primary caregivers, mostly the biological mothers, and the baseline characteristics of the caregivers are as shown in Table 1.

**Table 1:** Baseline characteristics of the study participants.

Variable	n	%
**A. Child characteristics:**		
**Age (years)**		
<10	67	46.3
≥10	77	53.5
**Sex**		
Male	81	56.3
Female	63	43.7
**Number of siblings**		
≤4	94	65.3
>5	50	34.7
**Duration of symptoms before presentation (months)**		
<3	33	22.9
3-6	62	43.1
>6	49	34.0
**Cancer type**		
Hemato-lymphoid	55	38.2
Solid tumor	77	53.5
CNS	12	8.3
**B. Caregiver characteristics:**		
**Sex**		
Male	59	41.0
Female	85	59.0
**Age (years)**		
<30	14	9.7
≥30	130	90.3
**Primary caregiver**		
Yes	124	86.1
No	20	13.9
**Relationship with the child**		
Mother	69	47.9
Father	53	36.8
Other relations	22	15.3
**Education level of caregiver**		
Primary or none	76	52.8
Secondary	48	33.3
Tertiary	20	13.9

CNS, Central nervous system.

### Wasting status at diagnosis and short-term clinical outcomes

Of the 144 pediatric participants, over one-fifth had been deemed to be wasted by the primary attending physician based on the visual analogue of visible wasting. Subsequent assessment based on anthropometric indices found that 57 (39.6%) of the children were wasted—over a half of whom had moderate wasting. Within the short-term follow-up during treatment, about one-fifth (20.8%) of the participants developed neutropenia, 13.9% had sepsis, and 14.6% died (Table 2).

**Table 2:** Wasting status at diagnosis and short-term (within six months) clinical complications.

Variable	n	%
**Visible wasting** ^*^		
Yes	32	22.2
No	112	77.8
**Anthropometric wasting** ^¥^		
Normal	87	60.4
Wasted	57	39.6
**Degree of wasting**		
Moderate	31	54.4
Severe	26	45.6
**Neutropenia**		
Yes	30	20.8
No	106	73.6
Unknown^€^	8	5.6
**Sepsis**		
Yes	20	13.9
No	113	78.5
Unknown^€^	11	7.6
**Status**		
Alive,	110	76.4
Death,	21	14.6
LTFU^§^	13	9.0

* As assessed by the attending clinician prior to enrolment into the study;

¥ Assessed by the investigators at enrolment into the study; only weight was taken, height and MUAC were missed;

€ Missed being assessed, because got lost to follow-up (LTFU) or died early, among other reasons;

§ 2 were lost to follow up after one month, 1 after two months, 3 after three months, 3 after four months, and 4 after five months.

### Comparison of visual analogue of wasting assessment with anthropometric-defined wasting

Use of a visual analogue of visible wasting correctly identified 25 of the 57 children with wasting and 80 of 87 children without wasting, giving a sensitivity and specificity of 43.9% and 92.0%, respectively. With regards to the degree of wasting, the sensitivity to detect children with severe wasting was higher, with a slightly lower PPV. On the other hand, the sensitivity of visible wasting in detecting children with moderate wasting was very low, with a low PPV and a high false negative rate (Table 3).

**Table 3:** Sensitivity and Specificity of visible wasting assessment approach.

Indices of wasting	True number of cases	Visible wasting analogue assessment			
		Sensitivity % (95% CI)	Specificity % (95% CI)	PPV (%) (95% CI)	Kappa k (95% CI)
**Overall wasting**	57	43.9 (30.7-57.6)	92.0 (84.1-96.7)	78.1 (60.0-90.7)	0.39 (0.24-0.53)
**Severe wasting**	26	73.1 (52.2-88.4)	89.0 (82.0-94.0)	59.4 (40.6-76.3)	0.57 (0.39-0.73)
**Moderate wasting**	31	19.4 (7.5-37.5)	77.0 (68.1-84.4)	18.8 (7,2-36.4)	−0.04 (−0.18-0.13)

**Fig 2:**
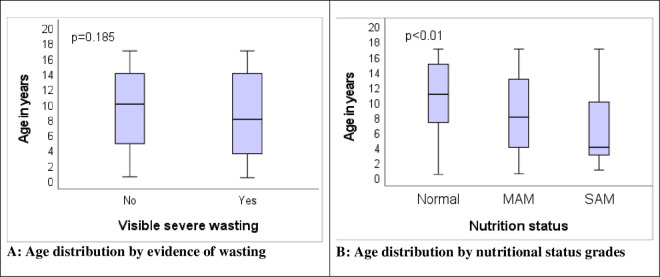
Box plots of the age distribution by wasting status.

There was no statistically significant difference in age between children and adolescents with and without visible wasting (p = 0.185; t-test) (Fig 2A). In contrast, children with cancer and anthropometric-defined severe wasting were significantly younger (median 4.0 years, IQR 3.0–10.7) compared to those with moderate wasting (median 8.0 years, IQR 4.0–13.0) and normal status (median 11.0 years, IQR 7.6–15.0) (p < 0.001; t-test) (Fig 2B).

#### Survival outcomes.

**Fig 3:**
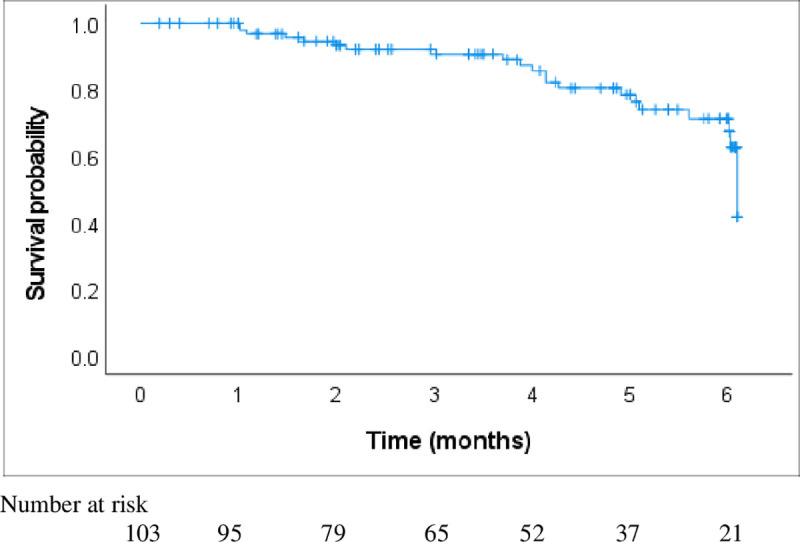
The probability of overall survival curves for children with cancer in the study cohort.

The short-term (six months) probability of overall survival (pOS) of the whole study cohort was 71.3% (95% CI 59.3–83.2) (Fig 3).

**Fig 4:**
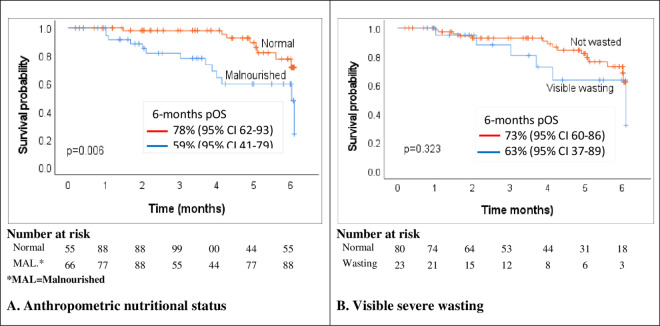
Overall survival curves for children with cancer by anthropometric and visible wasting status.

Fig 4 depicts the short-term overall survival among the childhood cancer patients by wasting status as determined by anthropometry and visual analogue. The pOS was significantly better for children with normal anthropometrically determined wasting status compared to those who are wasted (p = 0.006) (Fig 4A). For visible evidence of wasting, there was no statistically significant difference in the pOS between those noted to visibly severely wasted and their counterparts who were deemed not to be wasted (p = 0.323) (Fig 4B).

### Factors associated with wasting at diagnosis (anthropometric-defined and visible wasting)

#### Bivariate analysis.

In the bivariate analysis, there was a statistically significant association between wasting at diagnosis and the patients’ age and serum albumin. Children aged 10 years and above (adolescents) had a significantly lower occurrence of wasting at diagnosis compared to those aged less than 10 years. Likewise, children with lower serum albumin were 4.22 times more likely to be wasted compared to those with normal serum albumin. The relationship between the other explanatory variables and the outcome variables are as shown in Table 4.

**Table 4:** Relationship between patient characteristics and wasting status at diagnosis.

Variable	Visible wasting (n = 32)				Anthropometric wasting (n = 57)			
	%	OR	95% CI	*p*-value	%	OR	95% CI	*p*-value
**A. Child characteristics**								
**Age (years)**								
<10	26.9	1			52.2	1		
≥10	18.2	0.61	0.27-1.34	0.214	28.6	0.37	0.18-0.73	0.004*
**Sex**								
Female	20.6	1			39.7	1		
Male	23.5	1.18	0.53-2.62	0.686	39.5	0.99	0.51-1.94	0.983
**Number of siblings**								
0-4	22.3	1			41.5	1		
≥5	22.0	0.98	0.43-2.24	0.963	36.0	0.82	0.40-1.67	0.576
**Duration of symptoms**								
<3	18.2	1			42.4	1		
≥3	23.4	1.38	0.51-3.70	0.526	38.7	0.86	0.39-1.89	0.704
**Cancer type**								
Solid	22.5	1			43.8	1		
Hematological	21.8	0.96	0.43-2.17	0.927	32.7	0.62	0.31-1.26	0.187
**Serum albumin**¥								
Normal (≥35 mg/dL)	27.3	1			15.8	1		
Low (<35mg/dL)	72.7	2.67	0.65-10.89	0.172	44.2	4.22	1.46-12.18	0.008*
**B. Caregiver characteristics**								
**Age (years)**								
<30	14.3	1			42.9	1		
≥30	23.1	1.80	0.38-8.49	0.458	39.2	0.86	0.28-2.77	0.864
**Sex**								
Female	27.1	1			41.2	1		
Male	15.3	0.49	0.21-1.14	0.098	37.3	0.85	0.43-1.68	0.639
**Education level**								
Primary/none	27.6	1			42.2	1		
Post-primary	16.2	0.51	0.22-1.15	0.102	36.8	0.80	0.41-1.56	0.513
**Relationship**								
Mother	26.1	1			42.0	1		
Other relations	18.7	0.65	0.30-1.44	0.286	37.3	0.82	0.42-1.60	0.565
**Primary caregiver**								
Yes	21.8	1			38.7	1		
No	25.0	1.20	0.40-3.59	0.748	45.0	1.30	0.50-3.35	0.594

OR, Unadjusted Odds ratio; *p-value <0.05. ¥81 patients had serum albumin level estimates. The same model was used for both child and caregiver characteristics, but the results are presented under separate subheadings for clarity.

#### Association between wasting at diagnosis and short-term outcomes.

In bivariate analyses, wasting in children with cancer was statistically significantly associated with an increased risk of neutropenia, sepsis, and mortality. Visible signs of wasting was statistically significantly associated with increased risk of neutropenia and sepsis (Table 5).

**Table 5:** Bivariate analysis of association between wasting status at diagnosis and the short-term endpoints.

	%	Neutropenia			%	Sepsis			%	Mortality		
		OR	95% CI	p-value		OR	95% CI	p-value		OR	95% CI	p-value
**Anthropometric wasting**												
No	13.8	1			8.0	1			12.7	1		
Yes	36.7	3.63	1.56-8.42	**0.003**	28.3	4.50	1.65-12.29	**0.003**	31.0	3.08	1.15-8.28	**0.026**
**Visible wasting**												
No	16.0	1			9.7	1			18.5	1		
Yes	43.3	4.00	1.65-9.74	**0.002**	33.3	4.65	1.71-12.65	**0.003**	25.0	1.47	0.50-4.32	0.487

OR, Odds ratio; Number of participants included in the model for each outcome were 125 for Neutropenia, 122 for Sepsis, and 103 for Mortality.

#### Multivariate analysis.

On multivariate analysis, only low serum albumin remained a consistent predictor of wasting among childhood cancer patients (Table 6).

**Table 6:** Multivariate analysis of relationship between patient characteristics and wasting status at diagnosis.

Variable	Visible wasting (n = 32)			Anthropometric wasting (n = 57)		
	aOR	95% CI	*p*-value	aOR	95% CI	*p*-value
**A. Child characteristics**						
Age (≥10 years)	–			0.43	0.15-1.22	0.114
Cancer type (liquid)	–			0.48	0.17-1.39	0.177
Serum albumin§ (low)	2.67	0.65-10.89	0.172	4.22	1.46-12.18	**0.008**
**B. Caregiver characteristics**						
Sex of caregiver (male)	1.21	0.32-4.59	0.778	–		
Education level (post-primary)	0.52	0.14-1.98	0.341	–		

The multivariate model included variables with p values of ≤0.2 at bivariate level; aOR, Adjusted Odds ratio; §Serum albumin was available for 81 participants. The same model was used for both child and caregiver characteristics, but the results are presented under separate subheadings for clarity.

With respect to the short-term outcome endpoints, mortality was statistically significantly associated with both anthropometric and visible severe wasting. Neutropenia and sepsis also remained strongly associated with both measures of wasting, though these did not reach statistically significant levels (Table 7).

**Table 7:** Multivariate analysis of association between wasting status at diagnosis and the short-term endpoints.

	Neutropenia			Sepsis				Mortality	
	aOR	95% CI	*p*-value	aOR	95% CI	*p*-value	aOR	95% CI	*p*-value
Anthropometric wasting (Yes)	2.50	0.98-6.41	0.056	2.89	0.93-9.06	0.068	3.56	1.25-10.19	**0.018**
Visible wasting (Yes)	2.58	0.96-6.97	0.061	2.74	0.88-8.54	0.083	3.34	1.27-8.78	**0.014**

aOR, Adjusted odds ratio.

## Discussion

This study demonstrated that wasting is prevalent among our child and adolescent populations diagnosed with cancer, with increased risks of neutropenia, sepsis, and mortality. We have also demonstrated that the use of the subjective visual analogue of wasting is less reliable and not sufficient in determining the state of wasting for children with cancer.

The current study found a high frequency of wasting among children and adolescents newly diagnosed with cancer, which is consistent with that seen in many resource-limited settings in LMICs [31]. The 39.6% observed prevalence of wasting in our study is much higher than the current prevalence of wasting among children under five in Uganda [32], signifying the detrimental impact of cancer on childhood wasting. However, our observed rate is lower than the 63.3% found by Huibers et al. among children with cancer in Malawi [33], as well as other LMICs [7]. The observed disparities in the prevalence of wasting among children with cancer may not be surprising and could relate to the differences in the study population, cancer stage at diagnosis, and assessment methods used [11]. Nonetheless, the prevalence of wasting in our study population contrasts sharply with findings in HICs. For example, in a study at three tertiary care centers in Switzerland by Zimmermann et al., only 5.8% of pediatric patients newly diagnosed with cancer were wasted (or malnourished) [34]. The lack of a current clinical “gold standard” and consensus on the validity of the different parameters in children with cancer further adds to these differences [35,36]. Virtually all weight-dependent assessments have been shown to have shortcomings in children with cancer, particularly solid tumor with large masses and patients receiving steroid therapy [11]. Non-weight-based measures such as MUAC and TSFT have been considered to be more sensitive, identifying a higher proportion of children with wasting than BMI [37–39]. The presence of a large tumor mass, ascites, or edema, which can mask the effect of wasting and nutritional depletion on body weight, is believed to explain the observed limitations of weight-based measures [4]. In the setting of nutritional deficiencies, the nutritional reserves stored in the form of skeletal muscle protein and fat are depleted first, resulting in an early decline in MUAC and TSFT values [4].

The current study also demonstrated significant variation in the occurrence of wasting in relation to the child’s age. We found that younger children with cancer were more likely to be wasted than their older counterparts. This finding, though statistically significant only in univariate analysis, is consistent with findings among children in general in Uganda [40]. In particular, pediatric cancer patients with severe wasting were significantly younger than those with moderate wasting and normal status. In contrast, there was no statistically significant difference in age between children and adolescents with respect to visible evidence of wasting. This finding resonates with that by Li et al. in China, where undernourished – literally wasted, children had a significantly younger median age than the control group [35]. Our finding also supports an earlier finding in Uganda where younger age was a risk factor for wasting among children under five [40]. Nevertheless, age and wasting status among children with cancer have generally shown contradictory relationships. For instance, in their study cohort, Huibers et al. found a higher prevalence of acute malnutrition or wasting in the strict sense, in children older than five years. The authors attributed this observation to the fact that malnutrition guidelines in many LMICs do not prioritize children older than five, who often do not receive nutrition support [33]. The aforementioned differences notwithstanding, our finding seems to be in consonance with the fact that in LMICs, the risk of wasting is generally higher among children under the age of five years, with an increased risk of mortality [28].

In this study, we observed that many children and adolescents with cancer who had wasting would have been missed if assessments relied only on visible signs of wasting as an indicator of wasting. Compared to the anthropometric measure of severe wasting, visible wasting in the current study had low sensitivities in detecting wasting generally (43.9%) and moderate wasting (19.4%) specifically, with false negative rates of 56.1% and 80.6%, respectively. This finding is consistent with that reported in a study at two Kenyan hospitals where visible severe wasting failed to detect about half of the children with anthropometrically defined severe wasting, particularly in younger children [27]. In studies among Gambian nurses and Ethiopian health workers, visible wasting had relatively low sensitivities of 56% and 67%, respectively, immediately following training, compared to anthropometric measures [23,41]. In the Kenyan study, visible severe wasting had a sensitivity and specificity of 54% and 96% when assessed against MUAC <11.5 cm and 44.7% and 96.5% when assessed against WFH < −3 z-score, respectively [27].

The low sensitivity but high specificity of visible severe wasting for detecting children with anthropometrically defined wasting implies that visible severe wasting is highly selective and detects the most severely wasted children [27]. This was reflected in the current study, where the sensitivity of visible wasting was relatively higher for severe wasting but considerably lower for moderate wasting. The current findings have important clinical implications. The visible sign of wasting is subjective and mainly selects children at the extreme end of the wasting spectrum who are severely wasted. The subjective nature of visual indications of wasting stresses the importance of systematic clinical and anthropometric assessment in children with cancer based on weight, height, MUAC, and other non-weight-based parameters. As Cross et al. note, clinical examination in isolation of anthropometric measurements is inaccurate [42]. As a result, by relying solely on visible indicators of wasting, some wasted children – who may have nutritional deficiency, may remain unrecognized [43] and therefore miss out on the often-limited nutritional support available, increasing their risk of treatment-related complications and death [7].

The increased risk of treatment-related complications, notably mortality, but also neutropenia and sepsis, among cancer patients with wasting, may not come as a surprise, given that wasting—some of which is due to nutritional deficiency—has long been recognized as an adverse prognostic factor [10,11,44]. The increased incidence of neutropenia, which tends to be profound, prolonged, and associated with febrile episodes in the setting of wasting, has significant clinical implications, including delayed planned chemotherapy [10], prolonged hospitalization, increased need for supportive care, mortality, and reduced survival [45]. The low survival rates reported among cancer patients who are wasted [7,44], as also demonstrated in this study, especially in LMICs, could be attributed to treatment-related toxicities, compounded by the limited capacity for nutritional support interventions where this may be required. This therefore calls for action, including prioritized targeted nutritional interventions for high-risk patient groups, which, as evidence from Malawi [46] attests, can reduce morbidity and mortality. Likewise, as evidenced by a finding by Antillon et al. in Guatemala among children with acute lymphoblastic leukemia [47], improving the nutritional status of patients in LMICs with interventions results in experiencing less morbidity over time. Our study and those cited from the literature provide evidence that nutritional care is as essential to childhood cancer management as anti-neoplastic therapy, if not more so.

### Study limitations

One of the limitations of the current study was the inability to do a biochemical assessment of nutrition and micronutrient status, like serum pre-albumin, which is a more specific biomarker of nutritional deficiency that plays an essential role in the recovery of patients during management [48]. Likewise, the study relied on the primary attending clinician’s documentation of visible signs of wasting; however, it is possible that some children with wasting may not have been properly and systematically documented. The current study was also limited in the ability to determine the socio-economic background of the enrolled children, especially with respect to food security situation that could be used as proxy for distinguishing between wasting due to food scarcity and wasting due to cachexia process. Likewise, inadequate sample size for some important objectives (i.e., predictors of negative outcomes), impossibility to distinguish primary wasting and cachexia, absence of information on food security of the child’s household, and absence of information on treatment initiated, were the other limitations of this study. Nonetheless, this study derives its strength from being only one of very few studies that have assessed the use of clinical judgment to make a diagnosis of malnutrition in children with cancer in resource-limited settings. Being a prospective study following a rigorous protocol also adds to this strength.

## Conclusion and recommendations

Wasting at diagnosis is a common problem among children and adolescents with cancer in the study setting. Reliance on visual evidence of wasting as a marker for wasting only identifies children in the most severe stage of wasting. For accurate wasting categorization and management of those due to nutritional deficiency, all patients should undergo a standard anthropometric evaluation. Wasting among children with cancer is associated with an increased risk of mortality and other treatment-related complications, notably, neutropenia and sepsis. A comprehensive prospective study with a larger sample size is recommended. This should incorporate the assessment of biochemical parameters and the socioeconomic background with the assessment of household food security as a proxy for distinguishing between wasting due to food scarcity and that due to cancer cachexia.

## Supporting information

S1 File Sample size calculation. (PDF)

S2 File PLOS’ questionnaire on inclusivity in global research. (PDF)

S3 File Anonymized dataset. (XLSX)

S4 File Renamed_ba68a. (PDF)

S1 Checklist PLOS One clinical studies checklist completed. (PDF)

## Abbreviations:

BMI: Body mass index
CBC: Complete blood count
HIC: High-income country
IQR: Interquartile range
LMIC: Low- and middle-income country
MUAC: Mid upper arm circumference
PODC: Pediatric oncology in developing countries
SIOP: International society of pediatric oncology
TSFT: Triceps Skinfold Thickness
UCI: Uganda Cancer Institute
WFH/L: Weight-for-Height or Length
WHO: World Health Organization
