# Supplementary material for: Wasting and short-term outcomes among children with cancer in resource-limited settings: A prospective study in Uganda
Source: PLoS One. 2025 Aug 7;20(8):e0330107. doi: 10.1371/journal.pone.0330107 (PMC12331082; doi:10.1371/journal.pone.0330107)
Supplement: S1 File — (PDF) [file pone.0330107.s001.pdf]

» Sample Size Calculator

Sample Size Calculator (web)

Sensitivity/Specificity - Estimation

Expected sensitivity:	0.5	
Expected specificity:	0.95	
Prevalence of disease (p):	0.3	
Precision (± expected):	0.15	
Confidence level 100(1 - α):	95	%
Expected dropout rate:	0	%

Calculate

Reset

Sample size for sensitivity, n_{sen} =	143
Sample size for specificity, n_{spec} =	12
Final sample size (largest), n =	143
Final sample size (with 0% dropout), n_{drop} =	143

Formula reference:

Buderer, N. M. F. (1996). Statistical methodology: I. Incorporating the prevalence of disease into the sample size calculation for sensitivity and specificity. Academic Emergency Medicine, 3(9), 895-900.

Suggested reference:

APA: Arifin, W. N. (2025). Sample size calculator (web). Retrieved from http://wnarifin.github.io

Vancouver: Arifin WN. Sample size calculator (web) [Internet]. 2025 [cited 1 July 2025]. Available from: http://wnarifin.github.io

Sample Size Calculator by Wan Nor Arifin is licensed under a Creative Commons Attribution-NonCommercial-ShareAlike 4.0 International License.

© Wan Nor Arifin 2017-2025
