# Supplementary material for: Wasting and short-term outcomes among children with cancer in resource-limited settings: A prospective study in Uganda
Source: PLoS One. 2025 Aug 7;20(8):e0330107. doi: 10.1371/journal.pone.0330107 (PMC12331082; doi:10.1371/journal.pone.0330107)
Supplement: S2 File — (PDF) [file pone.0330107.s002.pdf]

STROBE Statement—checklist of items that should be included in reports of observational studies

	Item No.	Recommendation	Page No.	Relevant text from manuscript
**Title and abstract**	1	(*a*) Indicate the study's design with a commonly used term in the title or the abstract	1	We used a cross-sectional study design to collect quantitative data
		(*b*) Provide in the abstract an informative and balanced summary of what was done and what was found	2	The abstract of the report provides a summary and an explanation of what was done and the key findings of the study.
**Introduction**				
Background/rationale	2	Explain the scientific background and rationale for the investigation being reported	3	The scientific background and rationale of the study is explained in the background section of the report.
Objectives	3	State specific objectives, including any prespecified hypotheses	5	The purpose of this study was to evaluate the nutritional status of children and adolescents with cancer at the national cancer treatment centre in Uganda and determined the factors associated with malnutrition at diagnosis.
**Methods**				
Study design	4	Present key elements of study design early in the paper	5	We used a cross-sectional study design to collect quantitative data
Setting	5	Describe the setting, locations, and relevant dates, including periods of recruitment, exposure, follow-up, and data collection	5	The study setting was the paediatric oncology unit at the UCI in Uganda. UCI is a 200-

				bed national reference cancer treatment facility, 43 of which are dedicated to children and adolescent inpatients. Approximately 80% of children with cancer in Uganda are treated at the UCI, where about 400–500 new childhood cancer cases are seen annually, making this a representative site in the country for conducting the study.
Participants	6	(*a*) *Cohort study*—Give the eligibility criteria, and the sources and methods of selection of participants. Describe methods of follow-up<br>*Case-control study*—Give the eligibility criteria, and the sources and methods of case ascertainment and control selection. Give the rationale for the choice of cases and controls<br>*Cross-sectional study*—Give the eligibility criteria, and the sources and methods of selection of participants	N/A	
		(*b*)*Cohort study*—For matched studies, give matching criteria and number of exposed and unexposed<br>*Case-control study*—For matched studies, give matching criteria and the number of controls per case	N/A	
Variables	7	Clearly define all outcomes, exposures, predictors, potential confounders, and effect modifiers. Give diagnostic criteria, if applicable	8-9	The outcome variable was the proportion of children with cancer who were malnourished and who developed neutropenia, sepsis and mortality.
Data sources/measurement	8*	For each variable of interest, give sources of data and details of methods of assessment (measurement). Describe comparability of assessment methods if there is more than one group	*N/A*	
Bias	9	Describe any efforts to address potential sources of bias	N/A	
Study size	10	Explain how the study size was arrived at	6	A sample size of 144 participants was estimated using the single population proportion

formula by Kish Leslie based on a proportion (P) of 34.6%, with a marginal error (D) of 8%, a score in the standard normal curve(Z) corresponding to 95% certainty (1.96), and adjusted for potential dropout rate of 5%.

Continued on next page

Quantitative variables	11	Explain how quantitative variables were handled in the analyses. If applicable, describe which groupings were chosen and why	9-10	Descriptive statistics were used to summarize categorical variables as proportions and continuous variables as means (standard deviation) and median (interquartile range). Logistic regression with odds ratios and 95% confidence intervals were used to examine the association between the predictor and the outcome variables.
Statistical methods	12	(*a*) Describe all statistical methods, including those used to control for confounding	9-10	The report describes in detail the statistical methods used in the analysis of the reported data.
		(*b*) Describe any methods used to examine subgroups and interactions	N/A	
		(*c*) Explain how missing data were addressed	N/A	
		(*d*) *Cohort study*—If applicable, explain how loss to follow-up was addressed<br>*Case-control study*—If applicable, explain how matching of cases and controls was addressed<br>*Cross-sectional study*—If applicable, describe analytical methods taking account of sampling strategy	N/A	
		(*e*) Describe any sensitivity analyses	N/A	
Results				
Participants	13*	(a) Report numbers of individuals at each stage of study—eg numbers potentially eligible, examined for eligibility, confirmed eligible, included in the study, completing follow-up, and analysed	11	The number of individuals at each stage of the study is provided in the paper - summarized in a study flow diagram.
		(b) Give reasons for non-participation at each stage	N/A	
		(c) Consider use of a flow diagram		

Descriptive data	14*	(a) Give characteristics of study participants (eg demographic, clinical, social) and information on exposures and potential confounders	12	The report provides a summary of the characteristics of study participants.
		(b) Indicate number of participants with missing data for each variable of interest	12-18	The report indicates the number of participants with missing data for each variable of interest un the results section as a footnote for the respective tables.
		(c) *Cohort study*—Summarise follow-up time (eg, average and total amount)		
Outcome data	15*	*Cohort study*—Report numbers of outcome events or summary measures over time		
		Case-control study—Report numbers in each exposure category, or summary measures of exposure		
		Cross-sectional study—Report numbers of outcome events or summary measures	13-18	The report includes outcome events.<br>In the study population, 39.6% (57/144) of the children with cancer were acutely malnourished, 54.4% (31/57) of whom had moderate acute malnutrition and 45.6% (26/57) had severe acute malnutrition
Main results	16	(*a*) Give unadjusted estimates and, if applicable, confounder-adjusted estimates and their precision (eg, 95% confidence interval). Make clear which confounders were adjusted for and why they were included	16-18	The report gives both unadjusted and adjusted estimates under the results section
		(*b*) Report category boundaries when continuous variables were categorized	NA	
		(*c*) If relevant, consider translating estimates of relative risk into absolute risk for a meaningful time period	N/A	

Continued on next page

Other analyses	17	Report other analyses done—eg analyses of subgroups and interactions, and sensitivity analyses	N/A	
**Discussion**				
Key results	18	Summarise key results with reference to study objectives	19	This study demonstrated that malnutrition is prevalent among our child and adolescent populations diagnosed with cancer, with increased risks of neutropenia, sepsis, and mortality. We have also demonstrated that the use of the subjective visual analogue of wasting is less reliable and not sufficient in determining the nutritional status of children with cancer.
Limitations	19	Discuss limitations of the study, taking into account sources of potential bias or imprecision. Discuss both direction and magnitude of any potential bias	23	One of the limitations of the current study was the inability to do a biochemical assessment of nutrition and micronutrient status, like serum pre-albumin, which is a more specific biomarker of nutritional deficiency that plays an essential role in the recovery of patients during management. Likewise, the study relied on the primary attending clinician's documentation of visible signs of wasting; however, it is possible that some children with wasting may not have been properly and systematically documented.
Interpretation	20	Give a cautious overall interpretation of results considering objectives, limitations, multiplicity of analyses, results from similar studies, and other relevant evidence	19-23	The report gives interpretation of the results in line with the

				objectives and limitations of the study.
Generalisability	21	Discuss the generalisability (external validity) of the study results	23	The study was conducted in only one oncology unit in Uganda, and though the findings may not necessarily be generalizable to the whole region or other contexts, it is underscores the challenges that are prevalent in similar-resource settings.
**Other information**				
Funding	22	Give the source of funding and the role of the funders for the present study and, if applicable, for the original study on which the present article is based	24	This study did not receive any funding

*Give information separately for cases and controls in case-control studies and, if applicable, for exposed and unexposed groups in cohort and cross-sectional studies.

Note: An Explanation and Elaboration article discusses each checklist item and gives methodological background and published examples of transparent reporting. The STROBE checklist is best used in conjunction with this article (freely available on the Web sites of PLoS Medicine at http://www.plosmedicine.org/, Annals of Internal Medicine at http://www.annals.org/, and Epidemiology at http://www.epidem.com/). Information on the STROBE Initiative is available at www.strobe-statement.org.
